# Involvement of tumor necrosis factor-α in the upregulation of CXCR4 expression in gastric cancer induced by Helicobacter pylori

**DOI:** 10.1186/1471-2407-10-419

**Published:** 2010-08-11

**Authors:** Chenghai Zhao, Xiaomei Lu, Xianmin Bu, Ning Zhang, Wei Wang

**Affiliations:** 1 Department of Pathophysiology, College of Basic Medical Science, China Medical University, Shenyang, China; 2 Department of General Surgery, Shengjing Hospital, China Medical University, Shenyang, China

## Abstract

**Background:**

H. pylori, whose infection increases tumor invasiveness and metastasis, is generally labelled as the strongest risk factor for the development of gastric cancer. It appears not to be a coincidence that there is also an overexpression of CXCR4 and an obvious involvement in gastric cancer metastasis. The aim of this study attempts to investigate and further to establish a link between them. With H. pylori being a potent inducer of TNF-α, whether TNF-α, a tumor promoter, is involved in the induction of CXCR4 expression by H. pylori was also under research in this study.

**Methods:**

Expression of CXCR4, TNF-α, IL-6 and IL-1β mRNA was determined by real-time PCR. CXCR4 protein expression was detected by Western blotting. Concentrations of TNF-α, IL-6 and IL-1β in cell culture supernatants were measured using the Quantikine Elisa kit. To abrogate TNF-α expression in HGC27 cells, TNF-α RNAi plasmid was used to transfect them.

**Results:**

Levels of CXCR4 and TNF-α mRNA were significantly higher in H. pylori-positive gastric cancers (n = 19) compared to H. pylori-negative ones (n = 15). A subsequently Spearman's rank correlation test showed there was a positive correlation between the level of CXCR4 mRNA and that of TNF-α in 34 primary gastric cancers. Other results followed: Expression of CXCR4 and TNF-α was upregulated in gastric cancer cell MKN45 and HGC27 after infection with H. pylori 26695 (cag PAI^+ ^) or Tx30a (cag PAI^- ^); The induction of CXCR4 expression by H. pylori was inhibited significantly by a neutralizing TNF-α antibody, infliximab; CXCR4 expression was upregulated in MKN45 cells after treatment with exogenous TNF-α or co-culture with macrophage, and was downregulated in HGC27 cells after transfection with TNF-α RNAi plasmid. There was a significant increase in the migration of MKN45 cells treated with H. pylori 26695, and a strong inhibition when AMD 3100, a CXCR4 antagonist, or infliximab, was added.

**Conclusions:**

Our findings demonstrated that H. pylori upregulates CXCR4 expression in gastric cancer through TNF-α.

## Background

It is well accepted that Helicobacter pylori (H. pylori) is a strong risk factor for the development of various gastric diseases, namely chronic gastritis, peptic ulcers, gastric mucosa-associated lymphoid tissue lymphoma and gastric cancer, and it is acknowledged that the interaction between H. pylori and epithelial cells contributes to such development. In fact, H. pylori infection induces inflammation in microenvironment of the stomach associated with induction of proinflammatory cytokines, such as tumor necrosis factor-α (TNF-α), interleukin-1β (IL-1β) and IL-6[1-3], which makes gastric carcinogenesis conducive.

H. pylori infection also increases tumor invasiveness and metastasis [4-6], though the mechanism is still not well understood. The process of cancer metastasis is not random, and different cancers have their preferred homing sites. Just like leukocyte trafficking, tumor cell migration is critically regulated by chemokine/chemokine receptor system. Another focus of our attention is shed on CXCR4, the most common chemokine receptor overexpressed in a series of cancers (gastric cancer included) by far [7,8]. Studies have indicated CXCL12/CXCR4 axis is involved in gastric cancer metastasis [9]. Therefore it arouses great interests to find a link between H. pylori infection and CXCR4 overexpression in gastric cancer.

One of the key chemical mediators implicated in inflammation-associated cancers is TNF-α, and there is now substantial evidence in its involvement in promotion and progression of experimental and human cancers [10,11]. True to its name, high doses of regional TNF-α can lead to hemorrhagic necrosis via selective destruction of tumor blood vessels. However, it can unexpectedly act as an endogenous tumor promoter when produced in the tumor microenvironment. Our interest is consequently drawn to its involvement in the induction of CXCR4 expression by H. pylori, a potent inducer of TNF-α, which is known to upregulate a series of cytokines, chemokines, adhesion molecules and growth factors in cancers.

## Methods

### Gastric cancer cell lines and tissue specimens

The human gastric cancer cell MKN45 and HGC27 were obtained from Keygen Biotech. Co. (Nanjing, China), and were cultured in RPMI 1640 supplemented with 10% fetal bovine serum, at 37°C in a humid incubator with 5% CO_2_. 34 primary gastric cancer specimens were acquired from patients under operation with all their informed consent at Shengjing hospital, Chinese Medical University, and were frozen in liquid nitrogen immediately after surgical removal. Haematoxylin- and eosin-staining sections were prepared for assessment of the percentage of tumor cells, and only specimens with > 70% tumor cells were selected for analysis. This study was carried out with the approval of the ethical committee of China Medical University. All experiments were done at least three times.

### Macrophage cell line RAW264.7

The macrophage cell RAW 264.7 was provided by the American Type Culture Collection (Rockville, MD, USA), and was maintained in Dulbecco's modified Eagle's medium, supplemented with 10% fetal bovine serum, at 37°C in a humid incubator with 5% CO_2_.

### H. pylori strains

H. pylori strain 26695 (ATCC 700392, cag PAI^+ ^) and Tx30a (ATCC 51932, cag PAI^- ^) were offered by ATCC (Rockville, MD, USA). They were grown on sheep blood agar plates at 37°C under microaerophilic conditions. Bacteria were transferred after 48 h into Brucella broth containing 5% fetal bovine serum for 24 h. A multiplicity of infection of 100 was used in all studies.

### Real-time reverse-transcription PCR

Total RNA was isolated from tissues and cell lines by Trizol (Takara, Dalian, China) according to the protocol supplied by the manufacturers. cDNA was synthesized using Takara RNA PCR 3.0 Kit (Takara, Dalian, China) in a total volume of 10 μl, containing AMV reverse transcriptase, 0.5 μl; random 9 primer, 0.5 μl; 25 mM MgCL_2_, 2 μl; 10 × RT Buffer, 1 μl; dNTP mixture (each 10 mM), 1 μl; RNase inhibitor, 0.25 μl; RNA 1 μl; dH_2_O, 3.75 μl. Conditions for RT were: 30°C for 10 minutes, 42°C for 25 minutes, 99°C for 5 minutes, and 5°C for 5 minutes. Real-time PCR was performed using the LightCycler system together with the LightCycler DNA Master SYBR Green I Kit (Roche Diagnostics). The total volume is 20 μl, containing 25 mM MgCl_2_, 3 μl; 10 × Buffer, 5 μl; 10 μM forward Primer, 1 μl; 10 μM reverse Primer, 1 μl; LightCycler DNA Master SYBR Green I, 2 μl; cDNA, 2 μl; dH_2_O, 6 μl. Conditions for PCR were: 50°C for 2 minutes, 95°C for 10 minutes, and then 40 cycles of 5 seconds at 95°C and 20 seconds at 60°C. The housekeeping gene glyceraldehyde-3-phosphate dehydrogenase (*GAPDH*) was used as an internal control. Gene expression was quantified by the comparative CT method, normalizing CT values to *GAPDH *and calculating relative expression values. Primer sequences were provided by Takala (Dalian, China) as follows: *CXCR4 *forward, 5'-GAGGAAATGGGCTCAGGG-3', reverse, 5'-AGTCAGCAGGAGGGCAGGGA-3'; *TNF-α *forward, 5'-AGTGACAAGCCTGTAGCCC-3', reverse, 5'-GCAATGATCCCAAAGTAGACC-3'; *IL-1β *forward, 5'-CCACCACTACAGCAAGGG-3', reverse, 5'-GAACTGGGCAGACTCAAA-3'; *IL-6 *forward, 5'-CCTTCGGTCCAGTTGCCTTCT-3', reverse, 5'-GCATTTGTGGTTGGGTCA-3'; *GAPDH *forward, 5'-GGGAAACTGTGGCGTGAT-3', reverse, 5'-AAAGGTGGAGGAGTGGGT-3'.

### Western blotting

Cell lysates were prepared with sample buffer [50 mmol/L Tris-HCl (pH 6.8), 100 mmol/L DTT, 2% SDS, 0.1% bromophenol blue, and 10% glycerol] and were subjected to a 12% sodium dodecyl sulfate (SDS)/acrylamide gel. The proteins on acrylamide gel were transferred to a nylon membrane, which was blocked overnight (4°C in PBS with 0.1% Tween and 10% milk powder). Polyclonal antibodies for CXCR4 (Santa Cruz, CA, America), and the corresponding secondary antibodies (Santa Cruz, CA, America) were applied before immunoblotting. The human gene *β-actin *(Santa Cruz, CA, America) was used as an internal control. Blots were visualized with FX pro plus system (Bio-Rad) and quantified using Scion Image 4.03 software.

### RNA interference

TNF-α RNAi plasmid and nonsilencing control RNAi plasmid were purchased from Takala (Dalian, China). Cells were seeded into a 24-well plate at a density of 2 × 10^5 ^. On the following day, cells were transfected with TNF-α siRNA or Control siRNA using Lipofectamine 2000 (Invitrogen, United Kingdom) according to the manufacturer's instructions.

### Elisa for cytokines in cell culture supernatants

Concentrations of TNF-α, IL-1β and IL-6 in cell culture supernatants were measured using the Quantikine Elisa kit (Boster, Wuhan, China) according to the manufacturer's instructions. The sensitivity of the assay was 2 pg/ml for TNF-α, 4 pg/ml for IL-1β and 4 pg/ml for IL-6.

### Migration assays

The migration of cultured cells was assayed using Matrigel invasion chamber (24-well format, 8 μm pore; BD pharmingen). Cells (5 × 10^5 ^) were added to the upper chamber and medium supplemented with CXCL12 (100 ng/ml, Sigma) was added to the lower chamber. Migration assays were incubated for 18 hours at 37°C and 5% CO_2_. Migrated cells on the lower surface were stained using 1% toluidine blue after fixation with 100% methanol. For each transwell, the number of migrated cells in 10 medium power fields (× 20) was counted.

### Statistical analysis

Mann-Whitney *U*-test was used to compare mRNA expression between H. pylori-positive and H. pylori-negative tumors. Correlation between CXCR4 expression and TNF-α expression in gastric cancer specimens was analyzed using Spearman's rank correlation test. Expression of mRNA in gastric cell lines was compared using Student's *t*-test or one way ANOVA. Statistical analysis was carried out using SPSS version 11.0 (SPSS, Chicago, IL, USA). Difference was considered significant when *P*-value was < 0.05.

## Results

### Expression of CXCR4 and TNF-α mRNA in primary gastric cancers

**Figure 1:**
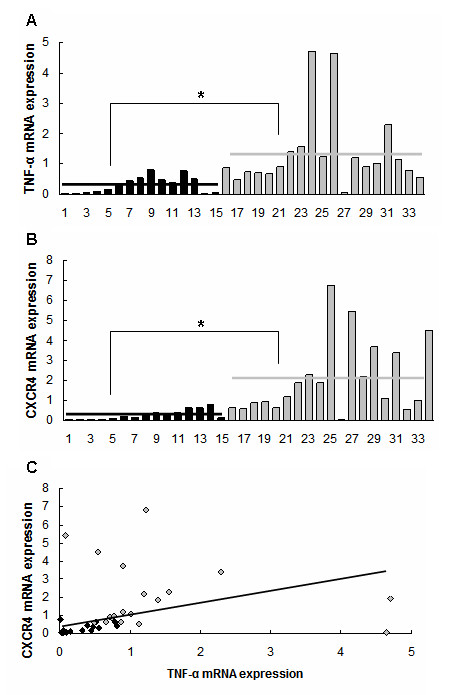
**Expression of CXCR4 and TNF-α mRNA in primary gastric cancers by real-time PCR**. (A) and (B) H. pylori-positive gastric cancers (grey columns, n = 19) expressed higher level of CXCR4 (A) and TNF-α (B) mRNA compared to H. pylori-negative gastric cancers (black columns, n = 15), *P < 0.01. Horizontal lines: means of mRNA level. (C) Level of CXCR4 mRNA was correlated positively with that of TNF-α mRNA in 34 gastric cancers, *P *< 0.01. Black rhombus: H. pylori-negative gastric cancers; grey rhombus: H. pylori-positive gastric cancers.

CXCR4 mRNA was determined by real-time reverse-transcription PCR in 34 gastric cancers, and its expression in each specimen was standardized to *GAPDH *expression. The results revealed its level significantly higher in H. pylori-positive gastric cancers (n = 19) compared to H. pylori-negative ones (n = 15) (7.2 fold, *P *< 0.01, Figure 1A). In the same cohort of tumors, TNF-α mRNA expression was also detected by real-time reverse-transcription PCR, and it was found stronger in H. pylori-positive gastric cancers compared to H. pylori-negative ones (4.3 fold, *P *< 0.01, Figure 1B). Spearman's rank correlation test further verified a positive correlation of CXCR4 expression with TNF-α in these 34 gastric cancers (*P *< 0.01, Figure 1C).

### Induction of CXCR4 and TNF-α expression by H. pylori in gastric cancer cells

**Figure 2:**
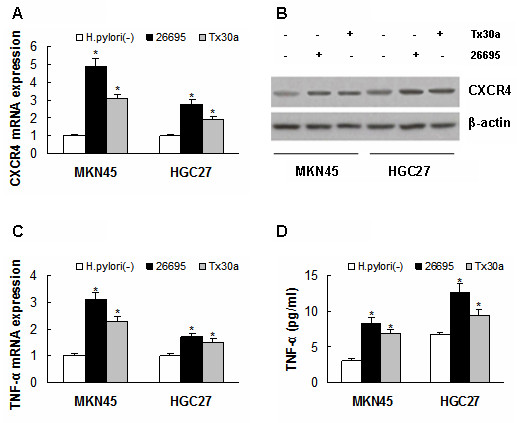
**Expression of CXCR4 and TNF-α in MKN45 and HGC27 cells after infection with H. pylori**. (A) and (B) CXCR4 expression was upregulated in MKN45 and HGC27 cells after infection with H. pylori 26695 (*P < 0.01, respectively, *vs *H. pylori^- ^) or Tx30a (*P < 0.01, respectively, *vs *H. pylori^- ^). (C) and (D) TNF-α expression was increased in MKN45 and HGC27 cells after infection with H. pylori 26695 (*P < 0.01, respectively, *vs *H. pylori^- ^) or Tx30a (*P < 0.01, respectively, *vs *H. pylori^- ^). Data are expressed as mean ± SD, n = 3.

Real time-PCR and Western blotting were used to detect CXCR4 expression in MKN45 and HGC27 cells (Figure 2A, B); and reveal it upregulated significantly in them after infection with H. pylori 26695 for 24 hours (*P *< 0.01, respectively, Figure 2A, B). Subsequently they were treated with a cag PAI-negative H. pylori, Tx30a to determine its involvement in the upregulation, and it is also found to induce CXCR4 expression in MKN45 and HGC27 cells (*P *< 0.01, respectively, Figure 2A, B).

The effect of H. pylori infection on TNF-α expression was further investigated using real-time PCR and Elisa, and the detection revealed that infection with 26695 or Tx30a for 12 hours may have led to both more expression of TNF-α mRNA (*P *< 0.01, respectively) and more secretion of TNF-α protein into the culture supernatant (*P *< 0.01, respectively) in MKN45 and HGC27 cells (Figure 2C, D).

### Effect of Infliximab, a neutralizing antibody to TNF-α, on the induction of CXCR4 expression by H. Pylori

**Figure 3:**
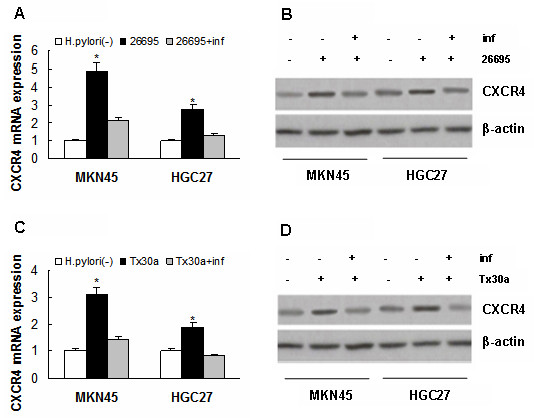
**Effect of infliximab on the induction of CXCR4 expression by H. Pylori**. (A) and (B) CXCR4 upregulation induced by H. pylori 26695 was inhibited in MKN45 and HGC27 cells after treatment with infliximab, *P < 0.01, respectively, *vs *26695 + inf. (C) and (D) CXCR4 upregulation induced by H. pylori Tx30a was also inhibited in MKN45 and HGC27 cells after treatment with infliximab, *P < 0.01, respectively, *vs *Tx30a + inf. Data are expressed as mean ± SD, n = 3. inf: infliximab.

The finding of upregulation of TNF-α expression in this case brought us to further research on its involvement in the induction of CXCR4 expression by H. pylori. A neutralizing TNF-α antibody, infliximab (4 μg/ml, Sigma), was then used to treat MKN45 and HGC27 cells after an infection by 26695, and the induction of CXCR4 was inhibited significantly (*P *< 0.01, respectively, Figure 3A, B). Similar results were observed when these cells were treated with infliximab after an infection by Tx30a (*P *< 0.01, respectively, Figure3C, D).

### Upregulation of CXCR4 expression in gastric cancer cells by TNF-α

**Figure 4:**
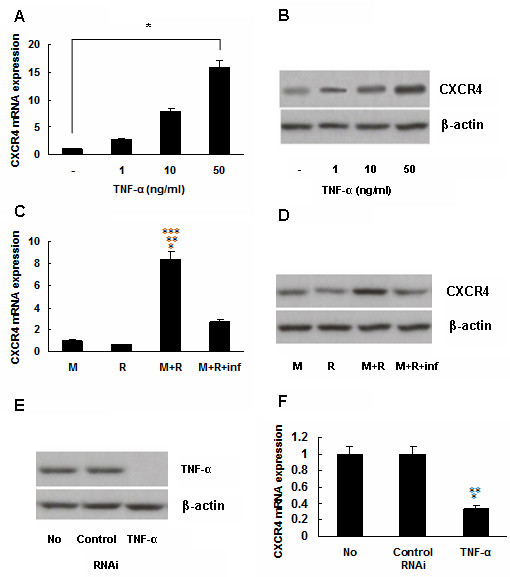
**Upregulation of CXCR4 expression in gastric cancer cells by TNF-α**. (A) and (B) CXCR4 expression was upregulated in MKN45 cells by exogenous TNF-α in a dose-dependent manner, *P < 0.01. (C) and (D) The coculture system of MKN45 and RAW264.7 cells expressed more CXCR4, *P < 0.001, *vs *M; **P < 0.001, *vs *R. The upregulation of CXCR4 expression was inhibited after treatment with infliximab, ***P < 0.001, *vs *M + R + inf. M: MKN45; R: RAW264.7; inf: infliximab. (E) Expression of TNF-α was absent in HGC27 cells after transfection with TNF-α RNAi plasmid. (F) CXCR4 mRNA expression was downregulated in HGC27 cells after transfection with TNF-α RNAi plamid, *P < 0.001, *vs *No RNAi; **P < 0.001, *vs *Control RNAi. Data are expressed as mean ± SD, n = 3.

MKN45 cells, which secret lower level of TNF-α protein, were treated with 1, 10, or 50 ng/ml TNF-α (Sigma) for 6 hours in attempt to further explore the role of TNF-α in the upregulation of CXCR4 expression, and real time-PCR and Western blotting detection revealed it was upregulated significantly in a dose-dependent manner (*P *< 0.01, Figure 4A, B), and even by 15.8 folds with 50 ng/ml TNF-α treatment.

Next, the CXCR4 expression within a co-culture system was examined, since tumor-associated macrophages also serve as a source of TNF-α in the gastric cancer microenvironment. A co-culture of MKN45 cells with RAW264.7 cells for 24 hours indicated it expressed significantly more CXCR4 mRNA and protein (*P *< 0.001, Figure 4C, D); this increase, however, was significantly inhibited by infliximab (*P *< 0.001, Figure 4C, D).

Finally, endogenous TNF-α was targeted to evaluate its regulation on CXCR4 expression in HGC27 cells, which secrets higher level of TNF-α protein. TNF-α RNAi plasmid was used to transfect HGC27 cells, and correspondingly nonsilencing RNAi plasmid was employed in the counterpart as control. It was observed that expression of TNF-α was absent in HGC27 cells after transfection with TNF-α RNAi plasmid (Figure 4E). Then expression of CXCR4 was detected by real-time PCR, and it was noted in the detection that, after transfection with TNF-α RNAi plasmid, CXCR4 mRNA expression was downregulated significantly in HGC27 cells, compared with cells with control RNAi or cells without RNAi (*P *< 0.001, respectively, Figure 4F).

### Induction of cytokines in macrophages by H. Pylori

**Figure 5:**
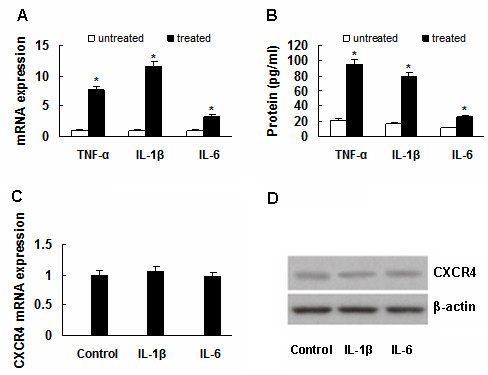
**Induction of cytokines in macrophages by H. Pylori**. (A) and (B) Expression of TNF-α, IL-1β and IL-6 was upregulated in 26695-treated RAW264.7 cells, *P < 0.001, *vs *untreated cells. (C) and (D) Neither IL-1β nor IL-6 can upregulate CXCR4 expression in MKN45 cells. Data are expressed as mean ± SD, n = 3.

Real-time PCR and Elisa were employed to evaluate the effect of H. pylori 26695 on cytokine expression in RAW264.7 cells, since it is generally acknowledged that cytokines are involved in chronic inflammatory processes caused by H. Pylori. The real-time PCR detection showed expression of TNF-α, IL-1β and IL-6 mRNA was upregulated in 26695-treated cells compared to 26695-untreated cells (TNF-α, *P *< 0.001; IL-1β, *P *< 0.001 and IL-6, *P *< 0.001, Figure 5A). And Elisa results indicated more proteins of TNF-α, IL-1β and IL-6 were secreted into culture supernatant in 26695-treated cells compared to 26695-untreated cells (TNF-α, *P *< 0.001; IL-1β, *P *< 0.001 and IL-6, *P *< 0.001, Figure 5B).

Finally MKN45 cells were treated with exogenous IL-1β (50 ng/ml) and IL-6 (50 ng/ml) respectively, to rule out the possibility that IL-1β and IL-6 may upregulate CXCR4 expression like TNF-α. Neither IL-1β nor IL-6 was found to upregulate CXCR4 expression significantly (Figure 5C, D).

### Migration analysis

**Figure 6:**
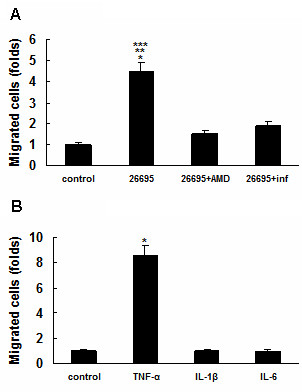
**Migration study.** (A) MKN45 cells showed a significant increase in their migration after treatment with 26695, *P < 0.001, *vs *control. The increase in migration of MKN45 cells induced by 26695 was inhibited when AMD 3100 (**P < 0.01, *vs *26695 + AMD), or infliximab (***P < 0.01, *vs *26695 + inf) was added. AMD: AMD3100; inf: infliximab. (B) TNF-α increased MKN45 cell migration, *P < 0.001, *vs *control. Neither IL-1β nor IL-6 can increase MKN45 cell migration significantly. Data are expressed as mean ± SD, n = 3.

Migration analysis was performed using the Matrigel invasion chamber in an attempt to investigate whether the upregulated CXCR4 was functional. In the initial phase of the experiment with 100 ng/ml of CXCL12 in the lower chamber, there was a significant increase (up to 4.5 folds) in the migration of MKN45 cells with 26695 treatment in the upper chamber, compared with control cells without 26695 treatment (*P *< 0.001, Figure 6A). Later on, however, the increase was inhibited remarkably when AMD 3100 (1 μg/ml, Sigma), a CXCR4 antagonist, or infliximab, was added (*P *< 0.01, respectively, Figure 6A).

In addition, the effect of cytokines on MKN45 cell migration was also examined, and TNF-α (50 ng/ml) was found to increase MKN45 cell migration significantly (*P *< 0.001, Figure 6B). However, neither IL-1β (50 ng/ml) nor IL-6 (50 ng/ml) was proved to promote MKN45 cell migration considerably (Figure 6B).

## Discussion

H. pylori is blamed to infect about 50% of the world's population as a definitive gastric carcinogen for humans [12]. Pathogenesis of its infection often includes inflammation, mucosal damage, or gastric atrophy, and requires close interactions between the bacteria and the gastric epithelial cells, activating signalling pathways, modifying host cellular functions, and leading to chronic epithelial responses [13.14]. There are now considerable evidences linking chronic inflammation to human cancers [15-17], and specifically, H. pylori-induced chronic inflammation and cytokines in local stomach microenvironment serve as the most common contributors [18-20]. This study highlights the result that mucosal level of TNF-α mRNA was significantly higher in H. pylori positive patients than that in negative patients by using quantitative real-time PCR, and two gastric cancer cells also secreted TNF-α protein in vitro. It further points to the assumption that TNF-α may be involved in H. pylori positive gastric carcinogenesis as an indispensable and strong linker between inflammation and cancer [21].

Though the mechanism of induction of TNF-α by H. pylori remains relatively unclear, a protein family has been disclosed in the last decade, including Helicobacter pylori-membrane protein-1 (HP-MP1) and TNF-α inducing protein (Tipα) [22-24]. Tipα gene, identified from H. pylori strain 26695, is homologous to HP-MP1 gene with 94.3% homology, and both of them show strong ability to induce TNF-α gene expression. In the study, H. pylori 26695 was found to upregulate TNF-α expression significantly in MKN45 and HGC27 cells, and cag PAI negative strain Tx30a also was spotted to induce it obviously, which may be in part due to the fact that HP-MP1/Tipα family is not in cag PAI region. However, it was also noted that the effect of Tx30a on TNF-α induction was weaker than that of 26695 (2.3 folds *vs *3.1 folds in MKN45 cells), which suggested H. pylori products in cag PAI may be also involved in the induction. In fact, it had been reported that cagA of H. pylori could induce TNF-α in gastric cancer biopsy specimens [25]. In addition, purified H. pylori urease was also found to induce MKN45 cells to express TNF-α [26].

TNF-α, a key cytokine in many chronic inflammatory diseases, was originally labelled as a serum factor for the induction of hemorrhagic necrosis of transplanted solid tumors in mice. However, presently it is commonly identified as a tumor promoter in local tumor microenvironment, and therefore the deletion or inhibition of it is supposed to reduce the incidence of experimental cancers. TNF-α/TNF-R1 knock down mice are resistant to chemical-induced carcinogenesis [27,28]. It is frequently detected in biopsies from a variety of human cancers, produced either by epithelial tumor cells or stromal cells. Additionally, it is also found, though in low amount, in the secretion of many cancer lines in vitro without inflammatory stimuli, though the mechanism is still not completely clear.

TNF-α was found not only involved in cell transformation and proliferation, but also in tumor metastasis. Such a finding was initially based on an animal model with colon cancer, in which injection of LPS enhanced the development of lung metastasis dependent on TNF-α production by host cells [29]. The subsequent results showed the increased tumor metastasis inhibited by neutralizing TNF-α antibody [30]. These led to our speculation that one of the underlying mechanisms of TNF-α in tumor metastasis may be related to the upregulation of chemokines/chemokine receptors. First, there was a significant upregulation of CXCR4 in gastric cancer cells after they were treated with exogenous TNF-α. There was another obvious upregulation of CXCR4 expression in cancer cells after they were co-cultured with macrophage, an alternative source of TNF-α in gastric cancer microenvironment. As was expected, this upregulation could be inhibited by TNF-α neutralizing antibody infliximab. There was consequently a remarkable reduction of the expression of CXCR4 in HGC27 cells after RNAi was employed to abrogate the TNF-α expression in these cells, which indicated endogenous TNF-α can also upregulate CXCR4 expression. The overall findings led to our conclusion that TNF-α, with itself involved in the metastasis of gastric cancer, upregulates CXCR4 expression.

Overexpression of CXCR4, whose involvement in various human tumors is well known, was frequently observed in gastric cancer tissues to increase gastric cancer metastasis. Some human gastric carcinoma cells also express CXCR4 mRNA and protein at high levels [7,8]. Our study showed H. pylori infection increased MKN45 cell migration through the upregulation of CXCR4 expression. The treatment with a CXCR4 antagonist AMD3100 resulted in a significant suppression of MKN45 cell migration in vitro. Another study showed AMD3100 significantly suppressed the development of peritoneal carcinomatosis in a mouse model of gastric cancer, which was evidenced by the reduction of tumor growth and ascitic fluid formation [9]. The previous researches led to our conclusion that CXCR4 overexpression in biopsy specimen of primary gastric cancer may serve as a preoperative evaluation of risks for the occurrence of peritoneal carcinomatosis.

Macrophages' involvement in the carcinogenesis and tumor invasion and metastasis [31,32] generally is blamed to motivate TAMs (Tumor associated macrophages), a major source of TNF-α in tumor microenvironment, to release a variety of growth factors, cytokines, and inflammatory mediators. The study revealed there was a significant expression of TNF-α induced by H. pylori, and simultaneously upregulation of IL-1beta and IL-6 in RAW264.7 cells, However, the latter variation failed to induce CXCR4 expression in MKN45 cells.

Studies have ultimately attributed the abnormal activation of NF-κB in cancer cells to the excessive secretion of TNF-α, whose role in CXCR4 upregulation is subsequently assumed to be related to pathways mediated by NF-κB. Others findings have revealed TNF-α antagonists can inhibit the upregulation of CXCR4 expression by H. pylori, and both it and CXCR4 antagonists can suppress the increased migration of gastric cancer cells in vitro. These results suggest that these antagonists alone, or in combination with other therapies, may serve as effective therapies for gastric cancer patients.

## Conclusions

TNF-α is involved in the upregulation of CXCR4 expression in gastric cancer induced by H. pylori.

## Competing interests

The authors declare that they have no competing interests.

## Authors' contributions

ZC designed the study, carried out PCR analysis, analyzed and interpreted the data, and drafted the manuscript. LX performed Western analysis. BM performed cell transfection. ZN was engaged in drafting the manuscript and in statistical analysis. WW performed Elisa analysis. All authors read and approved the final manuscript.

## Pre-publication history

The pre-publication history for this paper can be accessed here:

http://www.biomedcentral.com/1471-2407/10/419/prepub
